# Phospholamban and Sarcolipin Share Similar Transmembrane Zipper Motifs That Control Self-Association Affinity and Homo-Oligomer Stoichiometry

**DOI:** 10.3390/membranes16100323

**Published:** 2026-09-28

**Authors:** Bengt Svensson, Joseph M. Autry, John E. Rubin, Robyn T. Rebbeck, David D. Thomas

**Affiliations:** Department of Biochemistry, Molecular Biology, and Biophysics, University of Minnesota, 6-155 Jackson Hall, 321 Church Street S.E., Minneapolis, MN 55455, USAjer9173@med.cornell.edu (J.E.R.); rrebbeck@umn.edu (R.T.R.); ddt@umn.edu (D.D.T.)

**Keywords:** helix–helix interaction, heptad repeat, pore-forming peptide, sarcoplasmic reticulum, sequence design, transmembrane helix

## Abstract

We characterized the structural determinants of phospholamban (PLB) and sarcolipin (SLN) self-assembly, using experimental and computational assays. PLB and SLN are transmembrane peptides that regulate contractility via phosphorylation-dependent regulation of the sarcoplasmic reticulum Ca^2+^-ATPase (SERCA). The physiological role of PLB and SLN has been proposed to be important as a reservoir for fine-tuning SERCA activity and to act as ion-selective channels. Structural studies of PLB and SLN transmembrane domains have indicated stabilization of higher-order oligomers by leucine/isoleucine zippers in a heptad-repeat motif (a–g residue positions), requiring residue C41. Here we have modeled and tested the additional residues responsible for self-assembly using alanine replacement. We demonstrate that PLB e-position residues in the cleft between subunits contribute to self-association affinity and stoichiometry. For SLN, we identified two residues (V14, L21) and a novel heptad repeat (a-position) that contribute to self-association affinity and stoichiometry. This is in addition to the d-position that we previously identified for SLN oligomerization. Our molecular models demonstrate stable hexamer assemblies, but without the likelihood of a hydrated pore. We propose that PLB and SLN populate a distribution of oligomeric forms in sarcoplasmic reticulum membranes (monomer through pentamer and hexamer) and suggest that pore formation requires increased toroidal stoichiometry.

## 1. Introduction

Phospholamban (PLB) and sarcolipin (SLN) are transmembrane (TM) peptides that regulate Ca^2+^ transport of the sarcoplasmic reticulum Ca^2+^-transporting ATPase (SERCA), thereby regulating contractility in cardiac and skeletal muscle. The most common species-dependent expression is that PLB inhibits SERCA in ventricular and slow-twitch muscle, while SLN inhibits SERCA in atrial and fast-twitch skeletal muscle. β-adrenergic stimulation results in phosphorylation of PLB and SLN, thereby relieving SERCA inhibition and stimulating intracellular Ca^2+^ transport, with positive lusitropic and inotropic effects on contractility [1].

The monomeric forms of PLB or SLN bind to SERCA in a hetero-dimeric molecular complex (Ca^2+^ pump plus regulatory peptide), thereby inhibiting Ca^2+^ reuptake into the sarcoplasmic reticulum (SR) following intracellular Ca^2+^ release and myocyte contraction. Both peptides also self-assemble into higher-order homo-oligomers [2]. These regulatory peptides can also assemble into hetero-oligomers with each other in the tissue where they are co-expressed, adding another level of complexity to the regulation of SERCA activity [3]. Under physiological conditions, PLB exists in a dynamic equilibrium between pentamers and monomers, which is tuned by phosphorylation. This phosphorylation-dependent dynamic equilibrium controls the population of monomeric PLB available to actively inhibit SERCA activity [4,5,6,7,8,9,10]. Similarly, SLN exists in monomeric and oligomeric forms, whereby monomers actively inhibit SERCA activity [11,12,13,14].

The PLB monomer inhibits the Ca^2+^ affinity (1/K_Ca_) of SERCA, whereas the SLN monomer inhibits the Ca^2+^ affinity, maximal velocity (V_max_), and the Ca^2+^/ATP coupling ratio (Ca^2+^ ions transported per ATP molecule hydrolyzed) [1]. The functional roles of the oligomeric forms of PLB and SLN remain incompletely elucidated. Phosphorylated PLB pentamers serve as a reservoir pool of non-inhibitory subunits awaiting dephosphorylation and depolymerization to active SERCA-inhibitory monomers [13,15]. In vitro, non-phosphorylated PLB activates V_max_ of SERCA in reconstituted proteoliposomes at high PLB/SERCA ratios, and structural studies indicate binding of PLB pentamers to SERCA under these conditions [16]. For SLN, the active inhibitory species is monomeric [2,13].

There are multiple reports that PLB and SLN function as ion channels in vitro, presumably through oligomerization with toroidal geometry, with formation of a hydrated pore through the center of the oligomer (i.e., a barrel-like stave assembly) [17,18,19,20,21,22,23]. In vitro, PLB is a cation-selective channel (Ca^2+^ or K^+^) that is proposed to act in the SR (sarcoplasmic reticulum) as (i) a Ca^2+^ leak channel during relaxation, (ii) a Ca^2+^ release channel to help initiate contraction, or (iii) a K^+^ import channel to neutralize the large electrochemical gradient generated across the SR membrane during rapid Ca^2+^ release. In vitro, SLN is an anion-selective channel (Cl^−^ or PO_4_^2−^) that is proposed to act in SR as (i) a Cl^−^ export channel to neutralize the large electrochemical gradient generated across the SR membrane during rapid Ca^2+^ release, or (ii) a PO_4_^2−^ import channel to reduce ionized Ca^2+^ concentration in the SR lumen by forming Ca^2+^-PO_4_^2−^ precipitates during Ca^2+^ reuptake [12]. Expression of PLB or SLN in *E. coli* decreases bacterial cell viability, possibly due to formation of leak channels, disruption of membrane integrity, and/or inhibition of membrane enzyme(s) [13,24].

Structurally, it has been demonstrated that one face of the PLB transmembrane domain contains hydrophobic residues that form zippers for pentamer formation [5,6,25,26,27]. Previous homology modeling suggested that SLN also forms higher-order oligomers by using leucine/isoleucine zippers. SLN has been shown to predominantly exist in dimeric and trimeric states, but also populates a broader range of homo-oligomers [26]. Helical wheel analysis and sequence comparison have indicated that position I17-SLN is analogous to the I40-PLB zipper position, and that mutation of I17 to Ala (I17A) impairs the ability of SLN to form higher-order oligomers. Further, evidence has begun to link these zipper motifs with ion channel activity. SLN expression in bacteria also reduces cell viability, as had been previously observed with PLB expression. However, expression of the I17A mutant restores bacterial cell viability, suggesting that the I17A mutation disrupts a leucine–isoleucine zipper that stabilizes toxic WT-SLN ion channels in *E. coli* cells.

Homology modeling and MD stimulation have been used to study a model of the pentameric SLN channel in the membrane. Molecular dynamic simulations have predicted leucine–isoleucine zippers, including the I17 residue, which mediate SLN dimerization and oligomerization [14,28]. Potential mean force energy barrier values indicated permeability of hydrated Na^+^ and Cl^−^ ions through pentameric SLN. As the narrowest portion of the pore and highest energy barrier for either Na^+^ or Cl^−^ passage correlated with the hydrophobic L21 position, it was hypothesized this residue serves as the ion gate and that hydration expanded the pore size sufficiently to allow ion passage—a “hydrophobic gate”.

Improved understanding of how PLB and SLN oligomers form and what regulates their assembly is crucial in determining their physiological function. Much remains to be understood of the structural motifs for PLB and SLN oligomerization. Our molecular modeling and experimental data help define the specific TM residue interactions and zipper mechanisms involved in SLN and PLB self-assembly. Taking these insights into consideration with other known evidence, we propose a dynamic model of PLB and SLN oligomerization that is mediated through hydrophobic zipper interactions.

## 2. Materials and Methods

### 2.1. Molecular Modeling

Models of the PLB and SLN wild-type and mutant oligomer states were built using CCBuilder 2.0 (https://pragmaticproteindesign.bio.ed.ac.uk/builder/ (accessed on 8 June 2026)) [29]. CCBuilder is a tool that builds coiled-coil α-helix models based on peptide sequences. Models were built for the transmembrane region only, residues R25 to L52 of PLB and residues S4 to Q30 of SLN using the rabbit sequences. Oligomer states, i.e., tetramer, pentamer, and hexamers, were generated, where the oligomer radius, tilt and pitch angles were manually optimized to generate the lowest energy structural models. The interaction energy between helices was calculated by the BUDE force field implemented in the BUFF module of ISAMBARD [30]. The value of residues per turn of the α-helix in the models was used to predict backbone strain and models that deviated from being close to the optimal 3.6 residues per turn were rejected. The resulting PLB wild-type pentamer model closely matches the available NMR structures for the transmembrane region [27], which validates the method. The final model for the PLB pentamer has a geometry that allows for di-sulfide bonds between Cys36 and Cys41 to be formed. Molecular modeling and energy calculations were used to predict the relative effect of mutants and WT within the specific oligomeric states of PLB and SLN based on helix–helix interaction energies. Structure figures were created using VMD 1.9.3 (https://www.ks.uiuc.edu/Research/vmd/ (accessed on 8 June 2026)) [31].

### 2.2. Materials

Chemicals were purchased from Sigma-Aldrich (St. Louis, MO, USA). cDNAs encoding cyan fluorescent protein (CFP) and yellow fluorescent protein (YFP) were purchased from Clontech/Takara Bio (San Jose, CA, USA). DNA mutagenesis QuikChange kit was purchased from Stratagene/Agilent Technologies (La Jolla, CA, USA). The baculovirus transfer plasmid pAcSG2 was purchased from Orbigen (San Jose, CA, USA). BaculoGold baculovirus DNA was purchased from BD Pharmingen (Franklin Lakes, NJ, USA). *Spodoptera frugiperda* (Sf21) insect cells were purchased from Invitrogen/Thermo Fisher Scientific (Waltham, MA, USA). Bacterial expression plasmid pProEx HTc was purchased from Invitrogen. Laemmli-type, pre-cast SDS-PAGE gels were purchased from Bio-Rad (Hercules, CA, USA). Anti-PLB mouse monoclonal antibody (mAb) 2D12 was purchased from Affinity Bioreagents (Golden, CO, USA). Anti-tetra-His-tag mAb IgG1 was purchased from Qiagen (Germantown, MD, USA) (catalog # 34670: 0.2 mg/mL) and used at 1:1000 dilution. The membrane was blocked with 30 mg/mL bovine serum albumin in Tris-buffered saline at pH 7.4 for 1 h.

### 2.3. Oligomerization Assay of C41L-PLB Tetramer Mutants Expressed in Sf21 Insect Cells

PLB cDNA was cloned from dog left ventricle using RT-PCR [13], with a sequence the same as previously reported [32]. C41L mutagenesis was conducted to generate a PLB sequence that predominantly forms tetramers on SDS-PAGE [5]. Subsequent alanine scanning mutagenesis of TM domain residues L37, I38, L39, I40, L42, and I48 was performed to examine the effect on oligomerization. The cDNA of the C41L-PLB mutant and each of its alanine TM mutants were then expressed in Sf21 insect cells via baculovirus infection. SDS-PAGE was used to analyze oligomerization of WT-PLB, C41L-PLB, and TM mutants of C41L-PLB, as expressed in Sf21 cells. Prior to electrophoresis, gel samples were solubilized in 1.0% SDS for 5 min at 23 °C. Pre-cast 4–15% acrylamide Laemmli gels were run as per the manufacturer’s instructions (Bio-Rad). Immunoblotting was performed using anti-dog PLB mAb 2D12 and secondary antibody with chemiluminescence detection.

### 2.4. cDNA Cloning, Plasmid Construction, and Site-Directed Mutagenesis

SLN cDNA was cloned from rabbit fast-twitch muscle mRNA using reverse transcription–polymerase chain reaction [13], finding a cDNA sequence that was the same as previously described [33]. cDNA encoding CFP or YFP was then fused to the N-terminus of SLN cDNA using a restriction enzyme/ligation strategy to generate CFP-SLN and YFP-SLN cDNA [13,34]. To eliminate dimerization of CFP or YFP, residue Ala206 was mutated to Lys (A206K) [35]. Alanine mutagenesis of SLN TM residues Val14 or Leu21 was used to create CFP-V14A-SLN or YFP-L21A-SLN.

### 2.5. FRET Assay of SLN Fluorescent Fusion Proteins Expressed in Sf21 Insect Cells

CFP-SLN, YFP-SLN, CFP-V14A-SLN, and YFP-L21A-SLN proteins were expressed in Sf21 insect cells via recombinant baculovirus [7,8,13]. For FRET microscopy, 6.0 × 10^5^ cells were infected in 60 mm^2^ glass-bottom dishes with a multiplicity of infection of 2 viruses/cell for the donor construct (CFP-based fusion protein) and 3 viruses/cell for the acceptor construct (YFP-based fusion protein). FRET was measured 48 h post-infection, as described below (acceptor photobleaching and spectral unmixing).

FRET microscopy was used to assess the interaction between CFP- and YFP-labeled SLN constructs. The epifluorescence microscope, photobleaching protocol, and fluorescence quantification of CFP and YFP fusion proteins were the same as previously described [7,8,13]. Energy transfer efficiency was calculated as

FRET = 1 − (F_DA_/F_D_),

where F_D_ is the fluorescence of the CFP donor in the absence of the YFP acceptor, and F_DA_ is the fluorescence of the CFP donor in the presence of the YFP acceptor. The experiment was performed at least twice (n > 2) for all FRET pairs using 75 cells for CFP-SLN + YFP-SLN, 50 cells for CFP-SLN + YFP-L21A-SLN, and 53 cells for CFP-V14A + YFP-SLN. The data was fitted to the Hill equation using Origin 2015 (OriginLab, Northampton, MA, USA) to calculate K_d_ values. The FRET_MAX_ calculated for the WT interaction (0.577) was used in the fitting of the data for the SLN mutants.

### 2.6. Antibacterial Assay for SLN Multimeric State

We used *E. coli* to express SLN and CAT control protein and measured viability [13]. A total of 10 ng of the DNA was transfected into 50 µL of *E. coli* BL21(DE3) cells for 30 min on ice, cells were heat-shocked at 42 °C for 45 s, incubated on ice for 2 min, 950 µL of LB at 23 °C was added, and cultures were incubated at 37 °C for 30 min with shaking at 250 rpm. After this, 50 µL of the cells was plated on 1% LB-agar culture plates supplemented with 150 µg/mL ampicillin (LB-Amp plate). Plates were incubated for 16 h at 37 °C. The next day, one colony was picked for overnight culture in 5 mL LB with 150 µg/mL ampicillin (LB-Amp culture). After 16 h of shaking at 37 °C and 250 rpm, 50 µL of the overnight cultures was diluted 1:100, and then 50 µL was spread on 5 mL LB-Amp plates for induction of expression. After 2.5 h of culture at 37 °C at 250 rpm, 100 µL of cells was diluted 100-fold into 10 mL of ice-cold LB-Amp and placed on ice. SLN expression was induced by the addition of 0.6 mM IPTG, and then the non-induced cells were diluted 100-fold into 10 mL of ice-cold LB-Amp. A total of 100 µL of the cells was plated onto LB-Amp plates and incubated 18.5 h at 37 °C. Then, 2 h after induction, *E. coli* cells were diluted 100-fold twice, as before, and 100 µL of the cells was plated onto LB-Amp plates. Induced cells on plates were incubated for 16 h at 37 °C. Cells were counted on plates. Control cells expressed his-CAT. Cells were counted manually on each LB-agar plate.

## 3. Results and Discussion

### 3.1. Models for PLB Oligomeric Self-Assembly

To identify the residues responsible for switching between multimeric states of PLB, we modeled the structural states with WT and mutants and calculated the transmembrane helix interaction energy (Figure 1). The lowest energy model for PLB has a radius of 6.5 Å, an interface angle of 23°, and a pitch angle of 200° for the tetramer; a radius of 7.5 Å, an interface angle of 22°, and a pitch angle of 190° for the pentamer; and a radius of 9.2 Å, an interface angle of 25°, and a pitch angle of 190° for the hexamer. The radius increases as the number of units increases in the oligomer. The PLB tetramer model is a tightly packed bundle of helices. The pentamer model has a narrow pore that is lined by hydrophobic residues, suggesting that it is not likely to function as an ion channel. The hexamer has a pore that is wider, possibly wide enough to fit a lipid or a hydrated ion. The pore is still only lined with hydrophobic residues. As the radius and the pore size increase in the structural models as the number of units are added, it is possible that higher-order oligomers can function as ion channels.

The transmembrane residue C41 has been well established to contribute to the multimeric state, with the C41L mutation known to promote the tetrameric state. This is reflected in our modeling, as the lower interaction energy values for the tetrameric state are lower for C41L in comparison with WT (Figure 1B,C). By introducing alanine scanning to the C41L variant, we can distinguish unique roles that these other TM residues may contribute to multimeric state switching. As shown in Figure 1C, alanine variants for L37, I40 and I48 feature higher energy for all oligomer states relative to the C41L variant, likely promoting the monomeric state. In contrast, C41L-L39A has a lower energy for all oligomeric states, which demonstrates promotion of multimeric states at the expense of the monomeric state. The alanine variants I38A and L42 do not appear to shift the interaction energies for the multimeric states in comparison with C41L, thus suggesting that these residues are not involved in interactions in the interfaces of the multimers.

We expressed PLB in Sf21 insect cells and conducted scanning alanine mutagenesis of the C41L-PLB tetramer to characterize the role of TM residues in PLB oligomerization. WT-PLB expression in cardiac SR results in a mix of oligomers including dimers, tetramers, pentamers, and hexamers, as seen on the SDS-PAGE immunoblot (Figure S1, lane 1). The oligomeric states of PLB WT and mutants have been previously studied by SDS-PAGE and immunoblots using this very specific antibody, and it is well established that the major bands correspond to the pentamer and the monomer. For a recent example see Cleary et al. (2025) [36]. As previously shown, and in alignment with our computational modeling (Figure 1), C41L expression hinders pentamer formation and results in a predominantly tetrameric population (Figure S1, lane 2). Alanine mutations of L37 and I40 in C41L-PLB result in the absence of tetramers and reduction to primarily monomers (Figure S1, lanes 3 and 6), which aligns with the higher interaction energies we observed in our computational model (Figure 1). This also aligns with a previous study that demonstrated alanine substitution in WT background promoted monomers and increased the inhibitory effect of PLB on SERCA2a [6].

Alanine mutation of residues on the opposite face of the TM helix (I38, L39, and L42) does not disrupt tetrameric formation (Figure S1, lanes 4 and 5). In our computational modeling, we also observe no shift in interaction energy barriers for I38A and L42A; however, we anticipated that L39A would promote the hexameric state (Figure 1C). Curiously, I38A and L42A were shown to promote the multimeric state over the monomeric state and reduce the inhibitory function of PLB on SERCA2a [6]. There was a trend for L39A to be less inhibitory, but it was not significant [6]. The apparent discrepancy in the data and previous reports suggests complexity and differences between the multimeric states and their functional effects on SERCA activity. Another interesting difference between computational and experimental studies is that we observe that alanine mutation of I48 in C41L-PLB diminishes the tetramer population and restores pentamers (Figure S1, lane 8), while the prediction from the modeling was that oligomers were destabilized. The restoration of the pentamer state aligns with a previous study that demonstrated increased multimeric states and a lower inhibitory effect on SERCA2a by I48A in WT PLB [6]. These static molecular models may not explain all aspects of the oligomer interfaces and energetics. Side-chain flexibility and dynamics from molecular dynamics simulations might provide further insight into the cases like C41L-L39A and C41L-I48A where the modeling and experimental results do not correlate.

We propose that introduction of the C41L mutation, via conversion from a polar to apolar residue, provides increased hydrophobicity to an additional zipper arm (*e*-arm with C41L and I48), which, in addition to the *a*-, b- and *d*-arms, totals four zipper arms to stabilize C41L tetramers (Figure 2). While C41L may add hydrophobicity to strengthen *e*-arm zipper interactions, the added bulk of the leucine residue likely provides a sterically counteractive force that destabilizes pentamers. This is supported by the I48A mutation below C41L likely relieving this steric constraint and thus allowing “native” hydrophobic zipper interactions—which we propose to include the same four arms as the C41L tetramer—to again favor pentamer formation. Observation of these changes provides support for the helical wheel model of PLB and highlights the importance of leucine/isoleucine zippers as stabilizing motifs across multiple oligomeric PLB species.

Notably, previous experiments which truncated the polar upper-TM region of an engineered, water-soluble form of PLB found that this also served as a switch from pentamer to tetramer, which could suggest a greater role for polar forces in higher-order PLB oligomerization [37]. However, emerging research has also continued to demonstrate the prominent role of van der Waals forces in stabilizing peptide oligomers, and an engineered PLB-like molecule devoid of strongly polar residues has even been shown to stabilize as a pentamer [38], which we suggest is secondary to hydrophobic zipper interactions.

The discovery here of a “steric switch” capable of converting between a PLB tetramer and pentamer, taken in light of other evidence, provides insight into the mechanics of how zipper interactions control oligomeric order. Mutations of *a-* and *d*-arm residues provide the strongest evidence of disrupting higher-order oligomerization (Figure 2) and thus may be the points of strongest contact between monomeric units in higher-order oligomer formation. However, with an increasing oligomer number—i.e., from tetramers to pentamers—an increasing angle between monomeric units would require a shift in monomeric contact toward the *e*-arm. Accordingly, the steric bulk introduced at C41L on the *e*-arm likely provides a barrier to any expanding angle between monomers and restricts C41L-PLB to a tetramer. Interestingly, a hexameric form of PLB has been observed with the N34A mutation, a residue also lying on the *e*-arm [5]. With reduction in steric bulk on the *e*-arm from this mutation, hydrophobic zipper interactions maintain a greater angle between monomers, resulting in the preponderance of hexamers rather than pentamers. Together, these steric changes on the *e*-arm mitigate oligomeric order in a predictable manner in relation to how they limit or liberalize zipper interactions centered on the *a*- and *d*-arms, implicating zippers as motifs controlling oligomer stoichiometry (Figure 2).

There are several studies providing evidence that PLB pentamers regulate SERCA, including a crystal structure of SERCA interacting with a PLB pentamer and a demonstration that SERCA V_max_ is increased with a higher concentration of PLB pentamers [16,39,40]. The Young group has proposed that the PLB pentamer sits at the Ca^2+^ entry funnel of SERCA, and its basic residues may thus perturb the surrounding lipid bilayer, resulting in greater mobility of SERCA subunits and an increased turnover capacity [16]. Evidence suggesting functions of the PLB pentamer beyond direct regulation of SERCA have also been proposed, such as its capacity to attenuate PKA to delay the phosphorylation of the PLB monomer [41,42]. The balance in monomeric to pentameric states is important for cardiac response between rest and exercise, with the hyper stabilization of the PLB pentamer associated with dilated cardiomyopathy [36]. The standing paradigm of PLB and SLN self-assembly equilibrium has long been that PLB/SLN monomers inhibit SERCA but can pentamerize to become inactive [13,15]. While oligomerization may in part serve a storage function for monomers, we propose that the self-assembly equilibrium is more complex, with a fluid range of oligomers that include tetramers, pentamers, hexamers, and higher-order oligomers, where these oligomer species carry multiple important functions including SERCA regulation and ion channel activity (Figure 2). The mutations proposed here can be used as tools to resolve the role each multimeric state may have on SERCA function.

### 3.2. Models for SLN Oligomeric Self-Assembly

To get a better understanding of the multimeric states of SLN, we modeled the oligomeric structure of WT and mutants targeting the leucine/isoleucine zipper region. The models were optimized to minimize the transmembrane helix interaction energy. The lowest energy model for SLN has a radius of 6.7 Å, an interface angle of 19°, and a pitch angle of 200° for the tetramer; a radius of 7.8 Å, an interface angle of 18°, and a pitch angle of 210° for the pentamer; and a radius of 9.5 Å, an interface angle of 22°, and a pitch angle of 205° for the hexamer. The SLN oligomer models all show a slightly higher radius and pore size than the corresponding PLB models. Also, in comparison to the PLB oligomers, the SLN oligomers have more charged or polar residues at both ends of the TM region. These observations can possibly explain why expression of SLN in *E. coli* has been shown to be more disruptive than PLB expression for cell viability. As the radius and the pore size increase in the structural models as the number of units are added, it is possible that higher-order oligomers, such as pentamers and hexamers, can function as ion channels.

Based on the importance of PLB residues L37 and L44 for the multimeric state, equivalent transmembrane SLN residues V14 and L21 have been suggested to contribute to the multimeric state of SLN. By introducing alanine scanning to the region between V14 and L21, we aim to test which TM residues may contribute to multimer formation in SLN. As shown in Figure 3A, V14A shows a lower helix–helix interaction energy, i.e., more stable oligomeric states relative to WT. In contrast, I17A, I20A and L21A have a higher energy for all oligomeric states, which suggests promotion of monomeric states at the expense of the multimeric states. The V15A shows only the hexamer model has a noticeable difference in interaction energy compared with WT. The alanine variants L16A, T18A, and L19A do not appear to shift the interaction energies for the multimeric states in comparison with WT, thus suggesting that these residues are not involved in interactions in the interfaces of the multimers.

In previous experiments, the baculovirus system was used to express SLN as a fusion protein with green fluorescent protein derivatives tagged at the N-terminus—CFP-SLN and YFP-SLN [13]. As reported, confocal fluorescence microscopy indicates that CFP-SLN and YFP-SLN are localized to the ER when expressed in Sf21 insect cells, and fluorescence microscopy with acceptor photobleaching indicated a high degree of association between SLN molecules, i.e., that SLN self-associates in live cell membranes. Corroborating this data in a different method of measurement, fluorescence spectroscopy of cell homogenates with detergent dissociation used to disrupt SLN-SLN interactions in isolated Sf21 microsomes demonstrated similar FRET efficiencies (Figure 4A).

In this experiment, SLN mutants with alanine substitution to V14 and to L21 were similarly fused with green fluorescent protein derivatives tagged at the N-terminus (YFP-L21A and CFP-V14A), expressed in Sf21 cells, and FRET microscopy was conducted on the oligomeric interactions for each of these SLN mutants following acceptor photobleaching. Mean FRET was calculated and binding curves generated using FRET vs. acceptor expression (Figure 4A). The binding curve for interactions between CFP-SLN and YFP-SLN from the previous experiment is displayed for comparison. To accommodate for subtle shifts in expression levels of CFP- and YFP-labeled SLN between WT and mutants, we used sub-saturating CFP-SLN expression and mainly focused on the relative shifts in the K_d_ values. The mean FRET between CFP-V14A and YFP-SLN was 0.254 ± 0.009, a decrease of 0.084 from that between CFP-SLN and YFP-SLN, and K_d_ was 19.7 ± 1.2 a.u., a 2-fold reduction in binding affinity from a K_d_ of 8.07 ± 0.31 a.u. between CFP-SLN and YFP-SLN. This reduction in binding affinity indicates that V14 provides an important contribution to SLN self-binding affinity, and the decreased mean FRET that occurs with disruption of this residue alone indicates that SLN oligomerization may be dependent upon it. Mean FRET for interactions between CFP-SLN and YFP-L21A was not different from interactions between CFP-SLN and YFP-SLN, but K_d_ was 13.7 ± 0.8 a.u., thus also demonstrating a reduction in binding affinity. Therefore, the L21 residue also plays an important role in stabilization of oligomer formation.

FRET analysis from our previous study [13] demonstrated that mutation of the I17 residue, mapped on the *d*-arm of a proposed 3.5 residue/turn helix model, similarly impairs FRET efficiency. Adding V14 and L21 to this helical wheel analysis places them in the *a*-arm of this model (Figure 4B). The L21A model data supports the importance of this residue in the formation of oligomers. The case for V14A is not as clear, where the model does not predict the same results as shown in this experiment. This can be because valine is not a perfect residue in the hydrophobic zipper motif, which typically consists of leucines and isoleucines. Again, these static molecular models may not explain all aspects of the oligomer interfaces and energetics such as side-chain flexibility and dynamics. It has also been shown by molecular dynamics simulations of SLN in membranes that the zipper is not stably formed across all protomer–protomer interfaces in the oligomers [26]. These results add to a growing body of hydrophobic residues identified as important in the self-assembly of SLN and which we propose are stabilizing oligomers via leucine/isoleucine zippers on these two adjacent helical arms.

We previously monitored the multimeric state of SLN and PLB by virtue of their antibacterial effects on the number of *E. coli* colonies [13]. SLN expression in *E. coli* starkly reduced viability to 5%, with viability completely restored by I17A mutation [13]. The viability of *E coli* colonies is an indirect measure of the toxicity of the expressed peptides and could reflect other issues such as protein misfolding, non-specific stress or membrane disruption. Here, we used this approach with alanine scanning of SLN TM residues, comparing their expression with that of the control protein (chloramphenicol transferase, CAT). As previously shown in [13], SLN was found to decrease viable cell counts to 5% ± 1% compared to CAT. Mutation of SLN residues L16, T18 or V19 on the opposite face of the TM helix still resulted in cell death (Figure 5A). This is also predicted by the modeling of the L16A, T18A, and L19A SLN oligomers that does not appear to shift the interaction energies for the multimeric states compared with WT. However, the deleterious effect of SLN on *E. coli* viability was eliminated by alanine substitution to V14, V15, I17, I20 and L21 in the TM domain (Figure 5A). All these residues, except for V14, were predicted from the molecular modeling to have less favorable interaction energies in the oligomeric states than the WT SLN. The computational prediction for V14 again does not match the experimental results probably because the static minimized molecular models do not accurately represent the flexibility and dynamics of the peptides at room temperature. These SLN residues are analogous to residues L37, I38, I40, L43 and L44 in PLB, of which L37, I40, L44 are pentamer-stabilizing residues in the zipper. The mutation of V14 and L21 to alanine also decreased SLN-SLN binding affinity and self-assembly as measured by FRET.

Placing these antibacterial assay results in helical wheel analysis (Figure 5B) is again consistent with leucine/isoleucine residues in the *a*- and *d*-arms, which we propose are forming hydrophobic zipper interactions to stabilize the oligomerization of SLN ion channels. Although these experiments do not directly measure ion conductivity, we believe that the stark deleterious effect of SLN expression in *E. coli* may be explained by ion leakage through SLN channels embedded in, or oligomers disrupting, the bacterial cell wall membrane. Structurally, channel formation could be consistent with oligomer formation as the difference in lethality of the alanine mutations matches the oligomer-stabilizing hydrophobic residues involved in forming zippers.

### 3.3. Functional Relevance of PLB and SLN Self-Assembled Oligomers

If hexamer and greater high-order oligomers (n ≥ 7) of PLB are required to form Ca^2+^ channels, what is the physiological role of pentameric PLB, the oligomeric species with the highest abundance on gels and the one most studied? There are a number of studies providing evidence that PLB pentamers regulate SERCA, including a crystal structure of SERCA interacting with the PLB pentamer, and a demonstration that SERCA V_max_ is increased with a higher concentration of PLB pentamers [16,39,40]. The Young group has proposed that the PLB pentamer binds at the Ca^2+^ entry funnel of SERCA, and its basic residues may thus perturb the surrounding lipid bilayer, resulting in greater mobility of SERCA subunits and an increased turnover capacity [16]. Evidence suggesting functions of the PLB pentamer beyond direct regulation of SERCA have also been proposed, such as its capacity to attenuate PKA to delay the phosphorylation of the PLB monomer [41].

There is also a growing body of evidence for the presence of “chansporters”—complexes between two structurally unrelated but interacting channel and transporter proteins—throughout various physiological systems in the body. The neuronal K_v_7.2/7.3 potassium channel, for example, has been found capable of interacting with two different sodium-coupled neurotransmitter transporters, where the potassium channel may be enhancing sodium transport while reducing the depolarization effect from sodium influx [43]. Thus, it is possible that PLB and SLN channels interact with SERCA in chansporter-like complexes that coordinately interact to mediate SR ion fluxes.

The standing paradigm of PLB and SLN self-assembly equilibrium has long been that PLB/SLN monomers inhibit SERCA but can pentamerize to become inactive [13,15]. While oligomerization may in part serve a storage function for monomers, we propose that the self-assembly equilibrium is more complex, with a fluid range of oligomers that include tetramers, pentamers, hexamers, and higher-order oligomers, and where these oligomer species carry multiple important functions including SERCA regulation and ion channel activity.

## Figures and Tables

**Figure 1:**
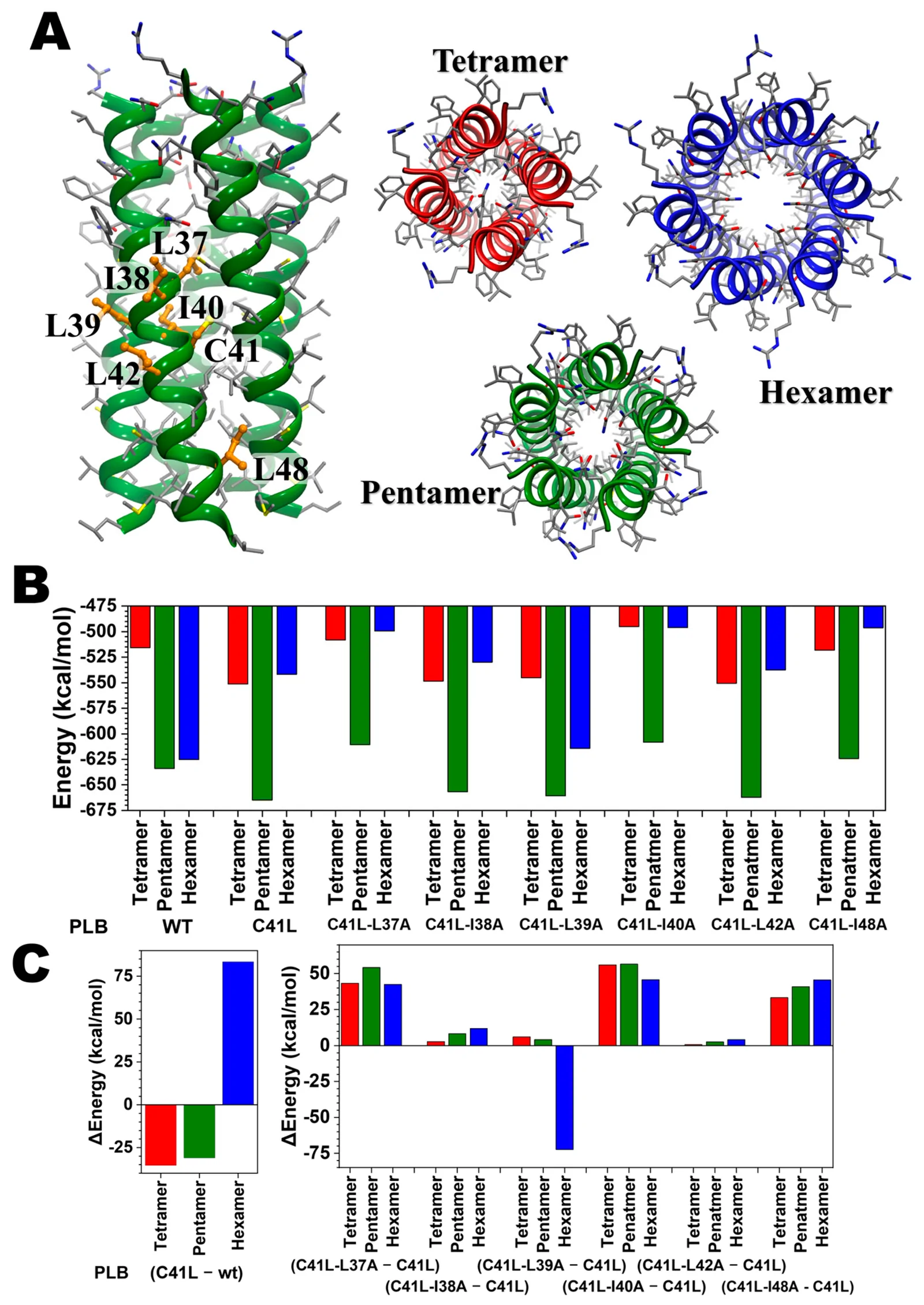
Modeling PLB self-assembly of tetra-, penta- and hexamers, with alanine substitution to resolve roles of TM residues in multimeric states. (**A**) The helical TM domain of PLB (residues R25 to L52) modeled as a tetramer (red), pentamer (green) and hexamer (blue). The seven residues that are the focus of this study are labeled in the pentamer. (**B**) For each multimeric state, interaction energies are plotted for the PLB variants wild-type (WT), C41L and double mutants C41L: L37A, I38A, L39A, I40A, L42A, I48A. (**C**) The difference (Δ) in interaction energy for each multimeric state for WT vs. C41L (left panel) and C41 vs. double mutants (right panel).

**Figure 2:**
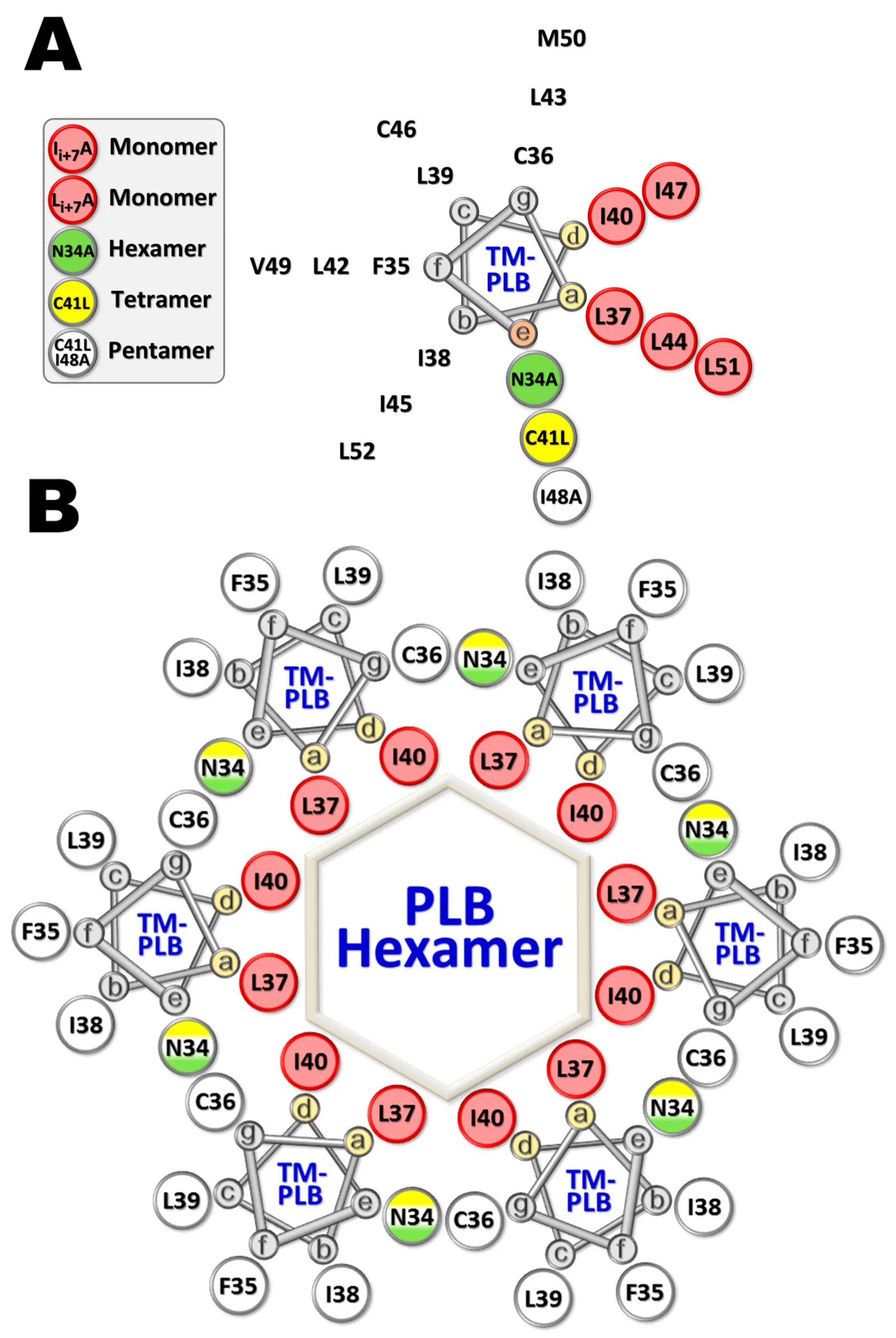
**Proposed models of hexamer formation.** (**A**) Residues 34–52 of PLB are modeled as a 3.5-residue/turn helix [5]. Cys41 was mutated to Leu (C41L) to probe oligomeric interactions of PLB. Isoleucine and leucine zipper residues are shown in red. (**B**) PLB hexamer.

**Figure 3:**
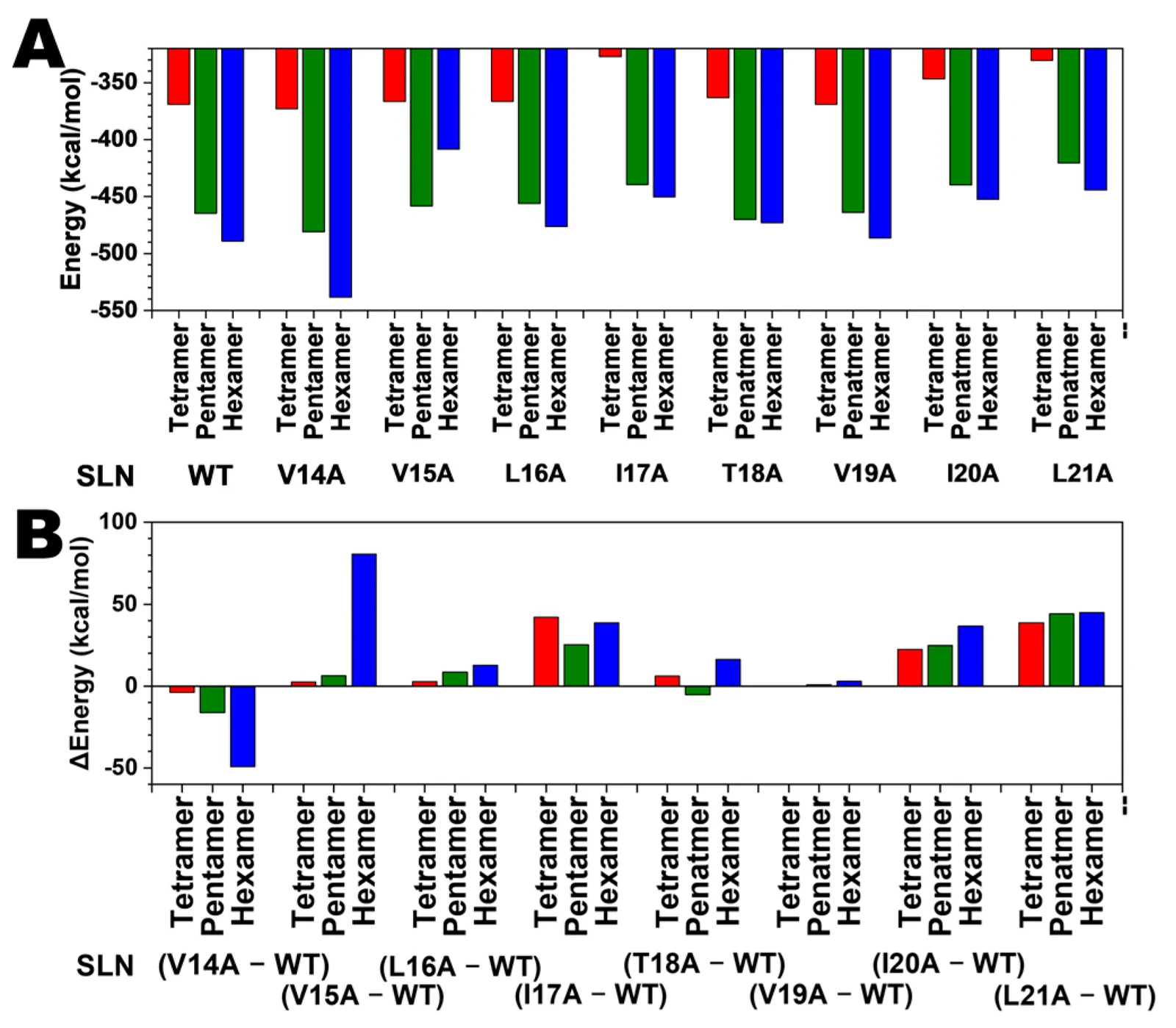
Modeling of SLN as tetra-, penta- and hexamers, with alanine substitution to resolve roles of TM residues in oligomer formation. (**A**) For each multimeric state (tetramer in red, pentamer in green, hexamer in blue), interaction energies are plotted for the SLN variants wild-type (WT) and the alanine variants V14A, V15A, L16A, I17A, T18A, V19A, I20A and L21A. (**B**) The difference (Δ) in interaction energy for each multimeric state for WT vs. alanine variants.

**Figure 4:**
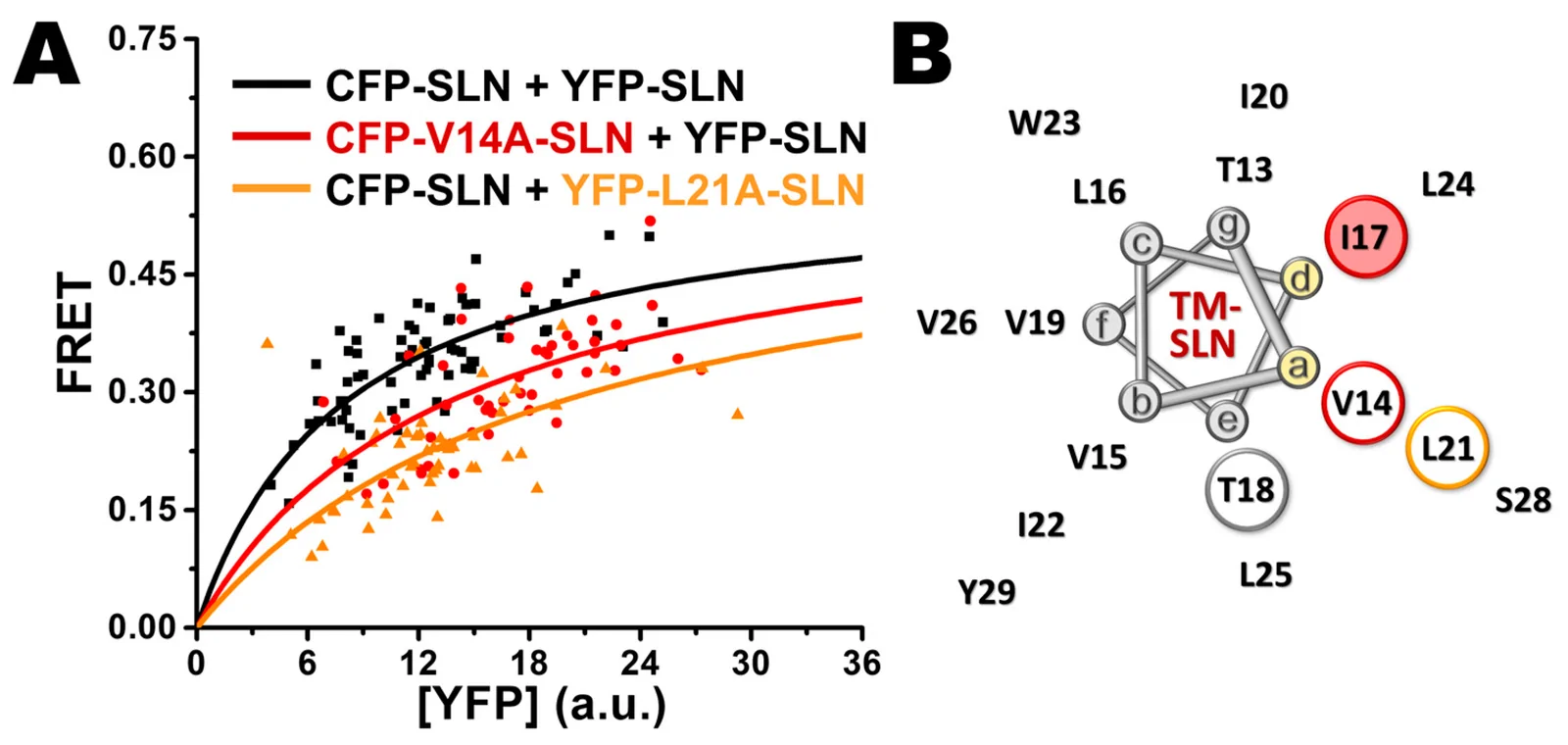
Zipper arm identification and control of SLN self-association affinity. (**A**) FRET binding curve. The experiment was performed at least twice (n > 2) for all FRET pairs using 75 cells for CFP-SLN + YFP-SLN, 50 cells for CFP-SLN + YFP-L21A-SLN, and 53 cells for CFP-V14A + YFP-SLN. The data was fitted to the Hill equation to calculate K_d_ values. (**B**) Results are mapped onto the TM domain of SLN modeled as 3.5 residue/turn helix. The T18A mutant (gray circle) on the *e*-arm (cleft position) results in a loss of channel activity in the planar lipid bilayer [12].

**Figure 5:**
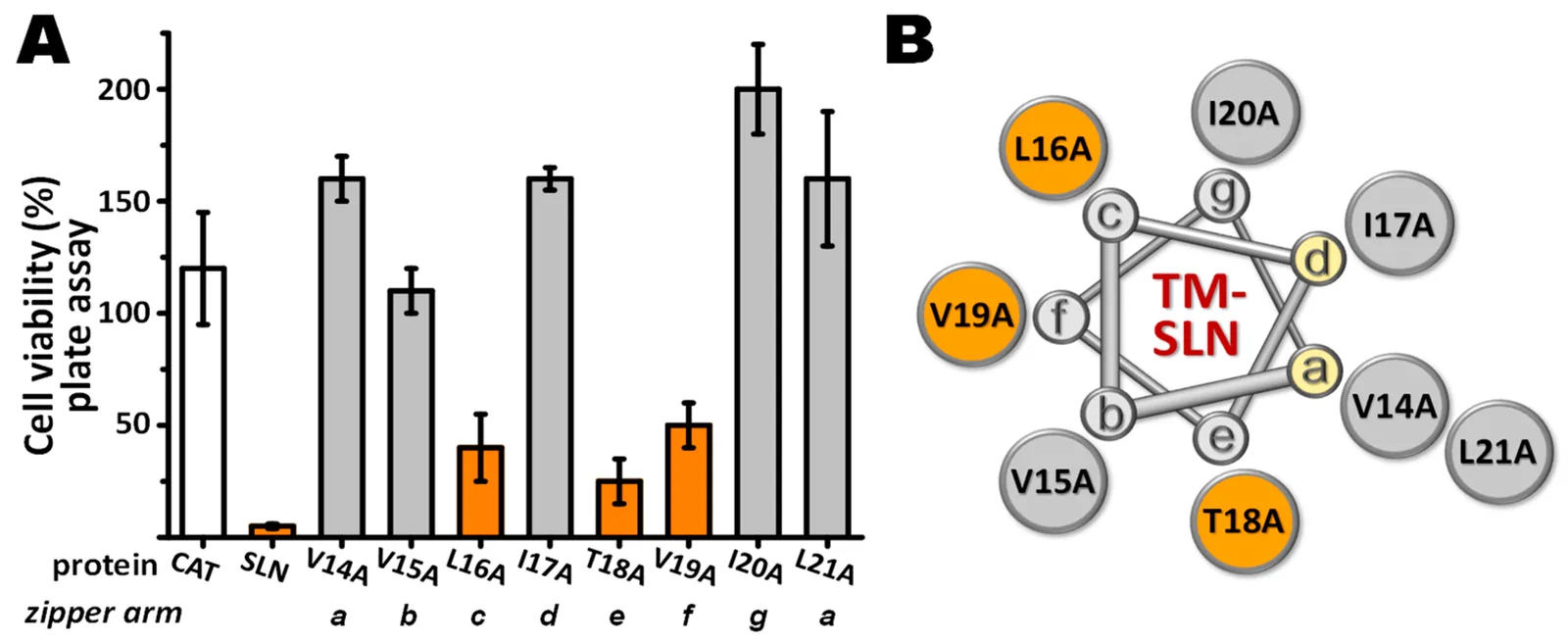
Effects of SLN expression and mutagenesis on bacterial growth. (**A**) Effect of Ala mutation on SLN antibacterial activity. Antibacterial experiments were performed three or more times (n ≥ 3). Error is reported as mean ± SEM. (**B**) Results are mapped onto TM domain of SLN modeled as 3.5 residue/turn helix. Residue positions that maintain antibacterial activity are colored orange, while residue positions that disrupt antibacterial activity are colored gray.

## Data Availability

The original contributions presented in the study are included in the article. Further inquiries can be directed to the corresponding author.

## Supplementary Materials

The following supporting information can be downloaded at https://www.mdpi.com/article/10.3390/membranes16100323/s1: Figure S1: Inter-subunit cleft residues help control the stoichiometry of PLB homo-oligomerization. Anti-PLB immunoblot of Sf21 homogenates expressing PLB proteins.

## Abbreviations

The following abbreviations are used in this manuscript:

CFP	Cyan fluorescent protein
FRET	Fluorescence resonance energy transfer
mAb	Monoclonal antibody
PLB	Phospholamban
SERCA	Sarco/endoplasmic reticulum Ca^2+^-transporting ATPase
Sf21	*Spodoptera frugiperda* insect cell line
SLN	Sarcolipin
SR	Sarcoplasmic reticulum
TM	Transmembrane
WT	wild-type
YFP	Yellow fluorescent protein
