# Supplementary material for: Phospholamban and Sarcolipin Share Similar Transmembrane Zipper Motifs That Control Self-Association Affinity and Homo-Oligomer Stoichiometry
Source: Membranes (Basel). 2026 Sep 28;16(10):323. doi: 10.3390/membranes16100323 (PMC13707140; doi:10.3390/membranes16100323)
Supplement: Supplementary file 1 [file membranes-16-00323-s001.zip › membranes-4520379-supplementary.pdf]

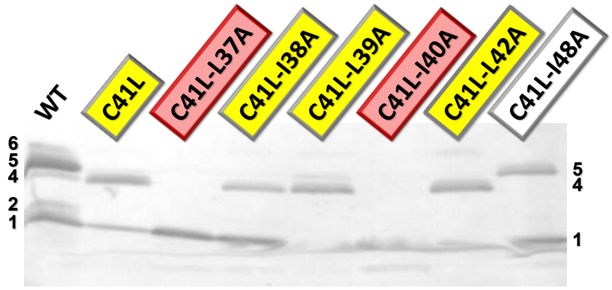

Figure S1. Inter-subunit cleft residues help control the stoichiometry of PLB homo-oligomerization. Anti-PLB immunoblot of Sf21 homogenates expressing PLB proteins. Labels indicate Ala mutants of C41L-PLB that are mostly monomeric (red), tetrameric (yellow), or pentameric (white).
